# LAITOR4HPC: A text mining pipeline based on HPC for building interaction networks

**DOI:** 10.1186/s12859-020-03620-4

**Published:** 2020-08-24

**Authors:** Bruna Piereck, Marx Oliveira-Lima, Ana Maria Benko-Iseppon, Sarah Diehl, Reinhard Schneider, Ana Christina Brasileiro-Vidal, Adriano Barbosa-Silva

**Affiliations:** 1 Genetics Department, Laboratório de Genética e Biologia Vegetal, Universidade Federal de Pernambuco, Recife, Pernambuco Brazil; 2 Queen Mary University of London, Centre for Translational Bioinformatics, William Harvey Research Institute, Barts and The London School of Medicine and Dentistry, Charterhouse Square, London, UK; 3 University of Luxembourg, Luxembourg Centre for Systems Biomedicine, Bioinformatics Core, Esch-sur-Alzette, Luxembourg

**Keywords:** Bioinformatics, PHP, Text mining, Soybean, *Arabidopsis thaliana*, Systems Biology

## Abstract

**Background:**

The amount of published full-text articles has increased dramatically. Text mining tools configure an essential approach to building biological networks, updating databases and providing annotation for new pathways. PESCADOR is an online web server based on LAITOR and NLProt text mining tools, which retrieves protein-protein co-occurrences in a tabular-based format, adding a network schema. Here we present an HPC-oriented version of PESCADOR’s native text mining tool, renamed to LAITOR4HPC, aiming to access an unlimited abstract amount in a short time to enrich available networks, build new ones and possibly highlight whether fields of research have been exhaustively studied.

**Results:**

By taking advantage of parallel computing HPC infrastructure, the full collection of MEDLINE abstracts available until June 2017 was analyzed in a shorter period (6 days) when compared to the original online implementation (with an estimated 2 years to run the same data). Additionally, three case studies were presented to illustrate LAITOR4HPC usage possibilities. The first case study targeted soybean and was used to retrieve an overview of published co-occurrences in a single organism, retrieving 15,788 proteins in 7894 co-occurrences. In the second case study, a target gene family was searched in many organisms, by analyzing 15 species under biotic stress. Most co-occurrences regarded *Arabidopsis thaliana* and *Zea mays*. The third case study concerned the construction and enrichment of an available pathway. Choosing *A. thaliana* for further analysis, the defensin pathway was enriched, showing additional signaling and regulation molecules, and how they respond to each other in the modulation of this complex plant defense response.

**Conclusions:**

LAITOR4HPC can be used for an efficient text mining based construction of biological networks derived from big data sources, such as MEDLINE abstracts. Time consumption and data input limitations will depend on the available resources at the HPC facility. LAITOR4HPC enables enough flexibility for different approaches and data amounts targeted to an organism, a subject, or a specific pathway. Additionally, it can deliver comprehensive results where interactions are classified into four types, according to their reliability.

## Background

In the past, scientific information used to be shared via letters between peers. This evolved to printed journals and magazines, and, during the early days of computation, diskettes became a popular way to exchange articles before the advent of the World Wide Web. Today, in the digital era, information has become more accessible, but it has also generated a new venture [1]. Likewise, keeping updated with the “state-of-the-art” and relating all the information available on most fields of study, if not all of them, have turned into an emerging challenge since the 21st century information boom in scientific publishing. According to NCBI resource coordinators (2018) [2], the number of full-text articles have been increasing at a rate of 11.35% a year!

To understand biology in all its complexity, it is necessary to comprehend the structure and dynamics of organisms from cellular to organismal levels. Thus, the focus must change from one element (e.g., protein, gene, phenotype) to a multidimensional point of view. Systems Biology approaches aim to access multi-OMICS data in a variety of experimental conditions and time series to exhaustively generate networks, which may offer an organism's response pathways overview under different situations [3, 4].

Research outcomes and relevant Systems Biology studies data are mostly reported in scientific journals [5]. The need for a more efficient way to explore the plethora of information buried in the various literature silos, has motivated the application of information retrieval and extraction techniques in biology. The area of text or literature mining has emerged, and it is expanding to fill the gap between published and useful information from scientific journals. Given the increase in articles’ availability and heterogeneity, text mining tools can boost the construction of new networks using pre-existing information, not to mention revealing insufficiently studied interactions of interest [6, 7]. Text mining can identify and extract biological entities co-occurrences in different levels, such as cellular, tissue and organism-specific contexts, allowing their integration in more informative networks [5, 8, 9].

Text mining tools follow three fundamental processes described by Krallinger and Valencia [10]: (i) information retrieval (finding relevant literature to be analyzed), (ii) biological entities (bioentities) identification (e.g., protein, gene, taxon tagging) and (iii) biological interaction terms to relate/associate the tagged entities. PESCADOR [9] is a web server based on LAITOR [8] and NLProt [11] text mining tools. It uses a list of articles identifiers (PubMed IDs – PMIDS) as a query to search and retrieve relevant abstracts. Furthermore, PESCADOR tags bioentities or biointeractions terms mentioned in the text collection (*corpus*) and identifies biological concepts and their co-occurrences along with bioentities. These co-occurrences are classified into four types according to their reliability, ranging from 1 (more likely to correspond to effective interactions) to 4 (less likely to correspond to effective interactions). Consequently, to build reliable pathways, manual curation is advised [8]. Type classification criteria are: (1) bioentity names co-occur in the same sentence with biointeraction term(s) between them; (2) bioentity names in the same sentence with biointeraction term in any position; (3) bioentity names in the same sentence, permissive identification of biointeraction terms; and (4) all biological entities of the abstract are retained (co-occurrence in the same sentence is not mandatory). Thus, co-occurrences of biological concepts are taken into consideration and reported for co-occurrences of types 1–4. Due to their complexity, the recognition of bioentities is usually the most time-consuming step. Consequently, making use of text mining approaches in big data has been a hard task.

Here, we propose a parallel, fast and unlimited text mining approach by adding customized programming functions suitable for HPC (high-performance computing). Text mining tools have been a valuable approach to support systems biology, not only for updating databases, but also for providing *ab initio* annotation of new pathways, by using automated text processing [12, 13]. To our knowledge, only STRING [14–17] has a programmatic version, but with a different approach than the one proposed here. STRING looks for co-occurrences based on a protein query with two text mining steps added after the update. On the other hand, LAITOR4HPC enables access to all entities ever described in a given species, as well as flexible keyword searches by naming a condition, a subject, or a specific protein, among other possibilities.

Three approaches were addressed to exemplify LAITOR4HPC's use cases. Firstly, all available abstracts of a selected species were analyzed, generating a report containing the absolute number of proteins, co-occurrences and interaction terms. The result provided enough data for stratification from most to least studied subjects. Secondly, considering a subject associated with biotic stress, 15 plants were analyzed to access the different levels of knowledge on a specific field, an approach that may enrich and generate pathways. Finally, a conceptual plant defensin (PDF) pathway is presented for *Arabidopsis thaliana*. PDFs are cysteine-rich, structurally conserved antimicrobial peptides, responsive to biotic stress, including bacteria [18], fungi [19] and insects [20, 21]. Besides the fact that PDF has previously been studied in many plants [21], most of the information about its regulation is scattered in the literature. Here we show the potential of LAITOR4HPC to gather comprehensive information on biological co-occurrences, allowing a conceptual and dynamic view of pathways.

## HPC parallelization and execution

All analyses were performed on the Gaia Cluster at Luxembourg University, High-Performance Computing Department. System configuration and cluster organization can be accessed online [22].

In the LAITOR4HPC version, abstracts must be provided as NCBI-PubMed XML format, and the files can be downloaded from PubMed server by doing a search using keywords, or accessed on MEDLINE FTP servers. A Python 2.7 script was written to parse the XML tree structure to recover the PMID, title and abstract of each record. The referred script was already updated to Python 3.0. The script provides an output, which is used as NLProt input [11], and the NLProt output is then used as LAITOR4HPC input.

To run the Python parser, we used the interactive (head) node, which is composed of Bull B500, 2 * Intel Xeon L5640 @ 2,26 GHz, 12 cores and 2880 Gb of RAM. Meanwhile, the following steps were run under the request of running nodes as described online. The NLProt step (bioentity tagging) analysis was launched as four distinct jobs, with 15 cores each (60 cores in total) and LAITOR4HPC was run as a single job, using a total of 20 cores.

### Parallelization

GNU Parallel software [23] was used to parallelize the analyses, with the flag “-j N”, where N represents the number of cores to be used, and each core is running the *i-th* input file at a time. To this end, a file containing the list of paths for all the input files was generated and shared across the cores to be used. Nevertheless, any tool with a similar function must work for the purpose of the LAITOR4HPC tool.

### Implementation

The time analyses and sources described concerned the first case study, since it was the most computationally intensive and time-consuming job. To run the time analyses, we have used all papers available until June 2017 from our selected corpus (i.e., MEDLINE), retrieved as previously described. A list with all PMIDs from our corpus is available in Supplementary Material 1. Then, the XML files for the corpus were parsed, and the parsed output was used as NLProt software input to highlight all bioentities (i.e., genes, proteins, taxon names, tissues and cell types). We used NLProt 1.0.2, made available by Rostlab [24]. The final step was to run LAITOR4HPC. LAITOR was initially developed using PHP [25] and its database was designed using MySQL database management system [8, 26]. LAITOR4HPC implementation is intended to be a stand-alone application, differently from LAITOR version which is integrated to PESCADOR, since jobs originated from web servers usually are executed in a dedicated (or virtual) machine, rather than in an HPC environment. Nevertheless, some of the newly implemented features can also run in a single core, such as the in-memory database query and the name tagging recovery.

A new optional step is to run the summary generator script. This script was written in Python3, with two running modes: (I) Basic: Generates N + 1 summary file, where N represents each LAITOR output in one folder and the extra file with an overall summary of all concatenated data. This is useful when a taxon is analyzed with different keywords searches, or when a big dataset is split for faster running; (II) Spread: Can check several folders to join all results of the basic summaries in a unique summary report. This is useful in cases where the same dataset is analyzed against many species.

All summaries inform how many proteins, co-occurrences and terms were targeted in the analyses. It is important to mention that the basic summary returns a text file for each LAITOR4HPC output, with the extension “.summary”. This file contains an ‘extra section’ describing all terms and how many times they were related to a given co-occurrence. Additionally, if only one file is available, the basic summary step will retrieve two very similar files. The additional file (+1) of the basic summary is named “A.join.dataset.summary” and does not contain the ‘extra section’.

**Fig. 1:**
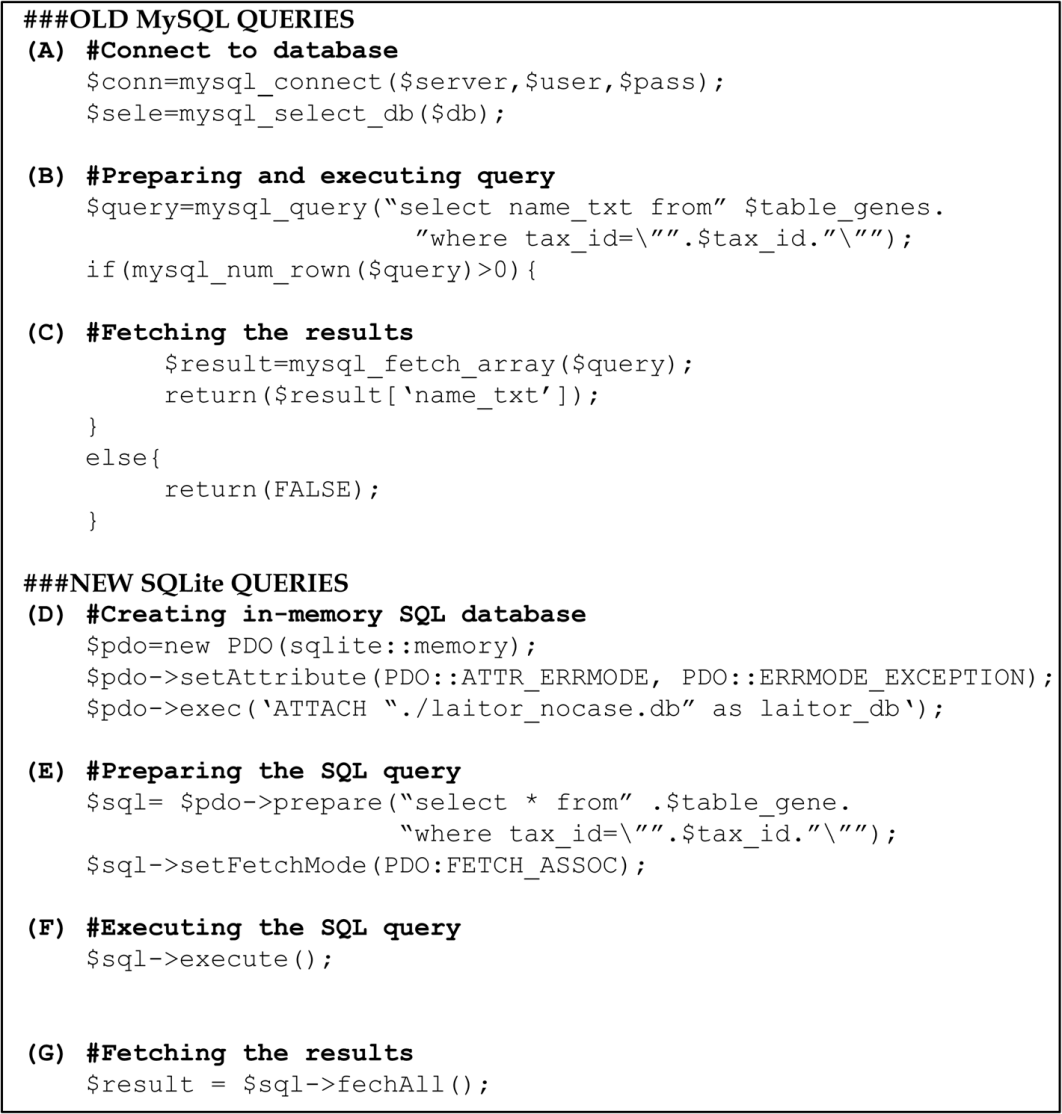
LAITOR4HPC database management system updates. The principles are the same as the previously online version. However, MySQL connects to a server where the database is stored in the disk **a**, whereas SQLite loads the database file in the RAM of the node executing the query **d**. The remaining processes are similar when using both technologies: **b, e, f** preparing and executing the query; and **c, g** retrieving the results

To distribute LAITOR as a parallel process, it was necessary to make sure that the processes running on different nodes could query the bioentities and biointeraction dictionaries seamlessly. However, MySQL requires its installation in every node for it to be used, which is possible, but against the user practices in most HPC systems, including ours. Therefore, we chose to switch the original disk-stored LAITOR databases (MySQL) by an in-memory database system. For that purpose, we used SQLite (version 3.0): a self-contained, highly reliable, embedded, full-featured, public-domain SQL database engine [27]. Consequently, we needed to adapt the queries from the former system to the latter (Fig. 1).

Three case studies were performed, aiming to encompass the different LAITOR4HPC applications. The first case study aimed to search all bioentities co-occurrences in all available abstracts for a given species (*Glycine max*); the second, to use keywords to look for all described interactions on one subject (biotic stress) in 15 different plant species; and the third, to build a pathway based on the information retrieved by the keywords “Plant AND Defensin” in *A. thaliana*.

For the first case study, the taxonomy identifier (tax-ID) filter option of LAITOR4HPC was used to check all soybean (*Glycine max* – Taxonomy ID: 3847) interactions described in 1134 XML files (approximately 30,000 abstracts each), comprising every MEDLINE paper available until June 2017. In this case study, no restriction on the subject was made. Therefore, all possible co-occurrences ever published about soybean could be retrieved. The basic summary report was used to access the 10 most studied proteins, co-occurrences and related terms.

In a second approach, a collection comprising a set of biotic stress-related keywords retrieved from MEDLINE (NCBI) was submitted to the pipeline 15 times, one for each plant species. This case study aimed to uncover interactions that are being over studied and some that are probably being ignored for some species. The basic summary report has evidenced the most studied proteins, co-occurrences and related terms, as well as the neglected ones for each species. Additionally, the ‘spread report mode’ has allowed the context analysis of each co-occurrence, independent of species specification.

For the third case study, the defensin-associated pathway regulation was built for *A. thaliana*. The parsed XML file related to the keywords “Plant AND Defensin” was selected. Furthermore, only interactions tagged as type 1 (proteins in the same sentence with the biointeraction term between them) were chosen to be used on CellDesigner [28] for construction of the pathway model. All the retrieved abstracts related to this step were manually curated, to verify possible false positives and interactions that may have not been tagged, thus allowing the expansion of the pathway beyond the automatic annotation (those interactions retrieved exclusively by the pipeline).

CellDesigner was used to make a conceptual visualization of the pathway by connecting the biointeraction terms with tagged proteins and reporting events of activation, regulation and inhibition. For a better visualization, different bioentities (genes, proteins and simple molecules) were represented by different shapes and colors.

#### Scalability test

The scalability test was performed using three XML files containing 1000 abstracts each, from three different species: *Caenorhabditis elegans*, *Homo sapiens* and *Arabidopsis thaliana* in a computer composed by an Intel Xeon(R) E-2124G CPU @ 3.40 GHz × 4 cores and 32 Gb of RAM following the same pattern proposed here to LAITOR4HPC.

First, we ran the Python parser script for each species using the GNU parallel. Then, the NLProt step was performed in two different stages: parallelized and non-parallelized, to evaluate the running time, per core usage and the number of tagged proteins. In the first case, the analysis was performed by running the three files using three, two and one core, sequentially. In the second run, the files were evaluated separately by using one core, but also a single file containing all the 3000 abstracts.

Finally, the LAITOR4HPC was carried out separately in one core to tag the interactions in each file and the running time, since the necessity of a specific tax-ID precludes the parallelization in this specific study case Table 1.

**Table 1:** Files, cores and their respective steps processed in each stage of the scalability test

No. files	No. Cores	Step
3	3	Parsing
3	3	NLProt
3	2	NLProt
3	1	NLProt
1	1	LAITOR4HPC (3x)

## Results

### First case study (soybean) and implementation

The first step, the Python parser script, was run on the head node against the 1134 XML files (approximately 31 M abstracts) in nearly 5 min. The second and third steps, comprised by NLProt and LAITOR, took 6 days in total to analyze all files filtering for soybean tax-ID. This represents an average processing rate of 0.017 s per abstract, which is a speed-up of approximately 117 times in comparison to the original implementation (in which the NLProt tagging alone took around 2 s to complete) [9]. The running time should vary depending on node configuration and cores available on the HPC, but it is faster than using a single core approach.

**Fig. 2:**
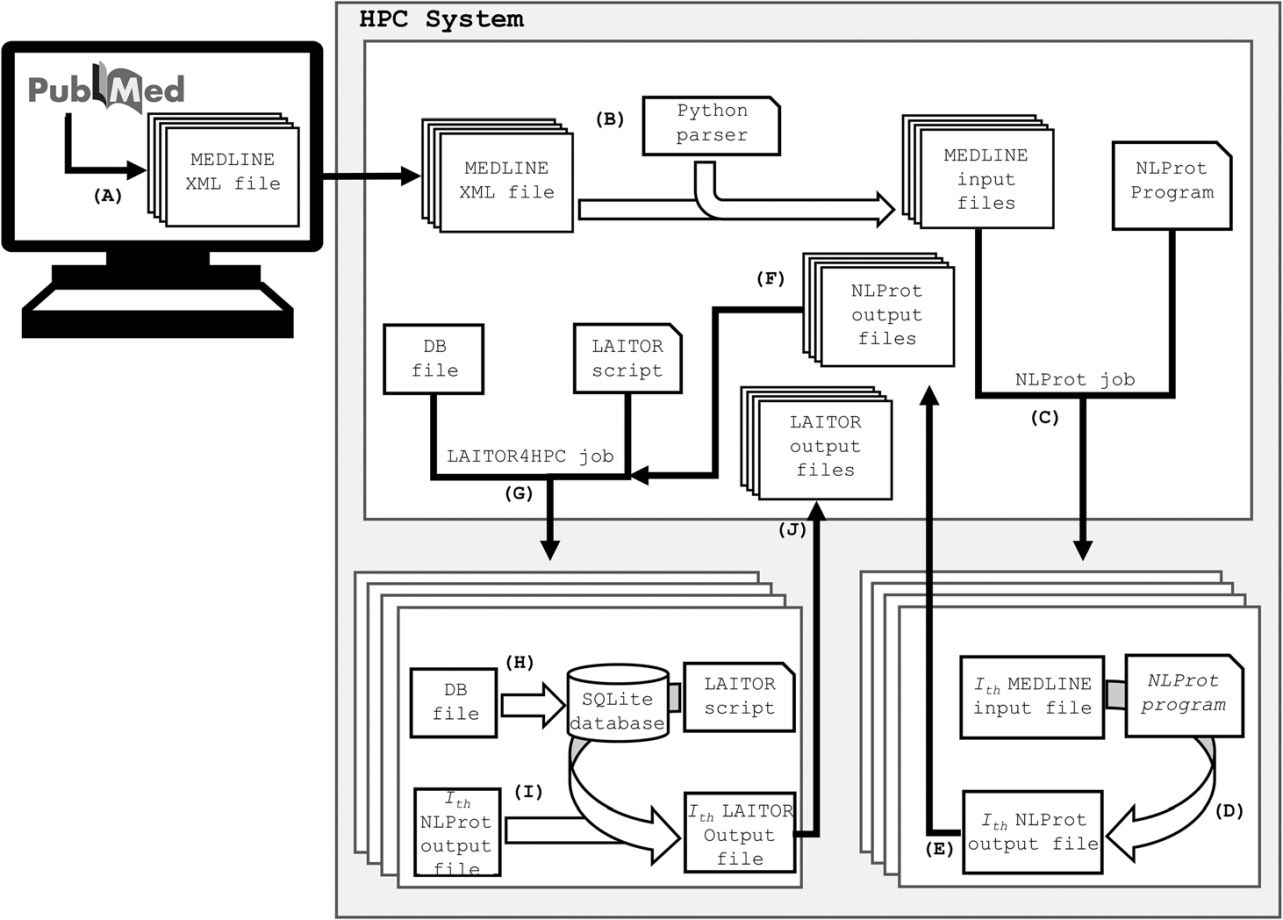
Complete text mining pipeline using NLProt and LAITOR4HPC. **a** MEDLINE files are downloaded from NCBI FTP as XML files; **b** a Python parser is executed to convert the XML files into input files for NLProt which are then **c** transferred into the interactive (head) node of the HPC system. **d** A job is then started and *i* different processes are launched in parallel on 60 computing cores (where: {*i* ∈ Ζ| {0 < *i* < 1305}). **e** In each core, the corresponding *i-th* MEDLINE input file is tagged by NLProt which generates **f** an *i-th* NLProt output file, which is then placed back to the head node together with the other outputs. **g** These files are used together with the DB file as input for the LAITOR4HPC job; **h** which loads an in-memory database before the **i** tagging of the bioentities and biointeraction present in the corpus. **j** After completion, the results are placed back to the head node and made available for downstream applications

Figure 2 represents the general pipeline obtained for the preparation of the MEDLINE abstracts as an input for the LAITOR4HPC text mining process. After downloading the full MEDLINE collection, a dataset of 1134 XML files was obtained, each containing approximately 30,000 PMIDS (Fig. 2a). These files were transferred to the HPC environment via SCP (Secure Copy Protocol) over an SSH (Secure Shell) protocol (Fig. 2b). The Python parser converted these records into readable NLProt MEDLINE input files (Fig. 2c). After that, the NLProt job was launched (Fig. 2d), where four nodes and a total of 60 computing cores were used to run *i* NLProt processes (where: {*i* ∈ Ζ | {0 < *i* < 1305}) to tag the bioentity names within those 1304 input files (Fig. 2e). Upon conclusion, those 1304 NLProt output files were made available in the head node (Fig. 2f), ready for the LAITOR4HPC step.

The LAITOR4HPC job execution uses the DB file and the NLProt output files as inputs (Fig. 2g). The jobs were launched from the head node, to be executed by one node with 20 cores. Each *i-th* process was directed to a corresponding computing core together with the DB file, the LAITOR4HPC script and the NLProt output. Every computing core loads the DB file as an SQLite in-memory database in that node during execution (Fig. 2h). Then the LAITOR4HPC script receives the *i-th* process and analyzes it against the loaded in-memory database, which contains the bioentity and biointeraction dictionaries (Fig. 2i). Once the results are obtained, they are made available back to the head node (Fig. 2j). At the end of the job, all the LAITOR4HPC output files are retrieved back to the head node and can be copied by SCP or another similar method to a user-client computer; from there, users can further explore the text mining outputs to create co-occurrence networks, for example.

By switching from MySQL to SQLite, we avoid HPC limitations during the database querying in the HPC architecture, as previously mentioned. Using SQLite in-memory, a new database is created purely in the memory of the computing nodes. This database ceases to exist as soon as the database connection is closed. As the database is self-contained in a text file, this file needs to be distributed across the computing cores along with the input file to be analyzed.

LAITOR4HPC running time was drastically decreased by the parallelization approach, which also allowed the user to query the whole corpus and extract all its bioentity co-occurrences. In comparison to the original version used by the PESCADOR website, where only a maximum of 1000 papers could be read on-the-fly, and NLProt alone was lasting 2 s. In LAITOR4HPC context: the more articles available, the better the result. Parallel SQL limitations caused by competitive accesses on the HPC environment were avoided by loading the database in the RAM of each computing node.

**Fig. 3:**
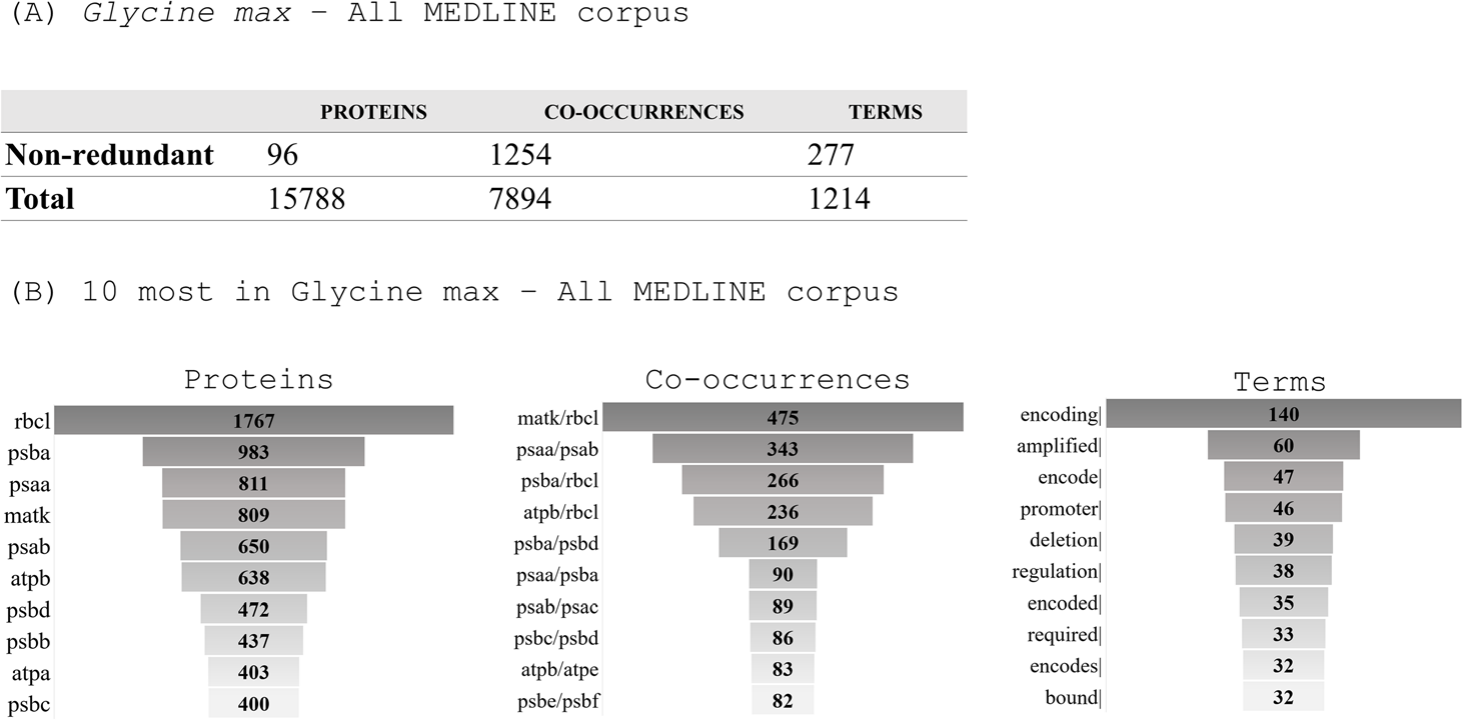
Mapping of LAITOR4HPC retrieved text analysis of all *Glycine max* available abstracts (until June 2017). **a** Non-redundant number of identified proteins, co-occurrences, and terms followed by absolute entities number. **b** Top 10 most described entities in absolute number. The list with all proteins, co-occurrences, and terms is available in Supplementary Material 2

After the soybean analysis performed on the overall MEDLINE corpus, the pipeline has tagged 15,788 proteins and 7894 co-occurrences along with the four occurrences types (type 1, 104; type 2, 685; type 3, 2369; type 4, 4736). The 96 non-redundant proteins were responsible for 1254 different co-occurrences in soybean. Rubisco Large subunit (rbcL) was tagged 1767 times and was present in three out of 10 most studied co-occurrences, followed by photosystem II protein A (psbA), which was tagged 983 times and present in four out of 10 of the most observed co-occurrences (Fig. 3), with 475 co-occurrences of Maturase K (matK)/rbcL. Not by chance, the most abundant interaction terms, among the non-redundant 227 terms, were *encoding* (140), *amplified* (60) and *encode* (47) (Fig. 3).

A closer look has revealed that all top 10 co-occurrences in soybean have at least one chloroplast-encoded protein related to photosystem II, and around 55% of mapped co-occurrences have the same pattern, with at least one of those being three chloroplast-encoded proteins (rbcL, psbA, matK) (Supplementary Material 2). The proteins, the co-occurrences and the terms that were only identified once or twice were considered as poorly described. Thus, for soybean, almost half of all the co-occurrences (751) and all the terms (128) were deemed as poorly characterized. All proteins, co-occurrences and terms can be found in Supplementary Material 2.

### Using keywords to search all described interactions on one subject

To describe the interactions related to biotic stress in plants, the same subset of papers was used to search for information about 15 species. The chosen keywords related to biotic stress were filtered for plants (keywords are listed in Supplementary Material 3) The first two steps (1) Python parsing and (2) NLProt tagging were run only once for all the analyses. The third step, LAITOR, was executed with different tax-IDs to specify the species. No co-occurrences were registered for three out of 15 species (*Medicago truncatula, Nicotiana benthamiana* and *Ricinus communis*).

**Fig. 4:**
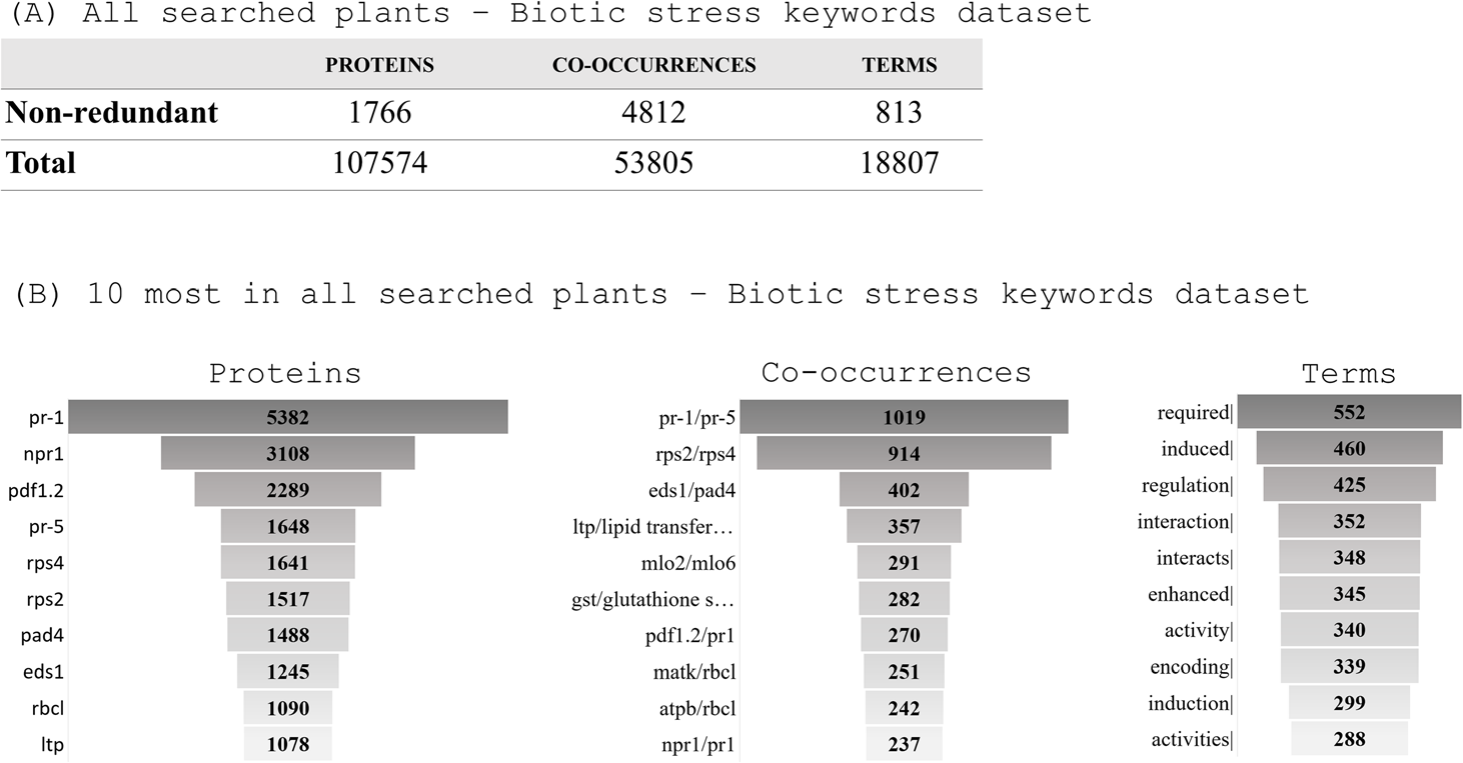
Mapping of LAITOR4HPC retrieved text analysis for 12 searched plant species among manuscripts subsets. The subsets were selected with 20 biotic stress-related keywords combinations (Supplementary Material 3). **a** Non-redundant number of identified proteins, co-occurrences, and terms followed by the absolute number of entities. **b** Top 10 most described entities in absolute number. The list with all proteins, co-occurrences, and terms is available in Supplementary Material 4

The number of proteins, co-occurrences and terms varied greatly among the remaining 12 species. Considering all tagged proteins, co-occurrences and terms (Fig. 4), PR-1 (Pathogen-related) protein family was explicitly the most widespread molecular entity (5382 descriptions and 138 unique co-occurrences), followed by NPR-1 (3108 descriptions and 173 non-redundant co-occurrences). PR-1/PR-5 and RPS2/RPS4 (ribosomal protein small subunit) were the most representative, with 1019 and 914 interactions, respectively. The profile of the most annotated proteins suggests that for the biotic stress-related subject, expression of responsive genes is the main focus of study, since among the most retrieved terms are: *required* (552), *induced* (460), *induction* (299), *enhanced* (345) (complete list of proteins, co-occurrences and terms available in Supplementary Material 4).

**Fig. 5:**
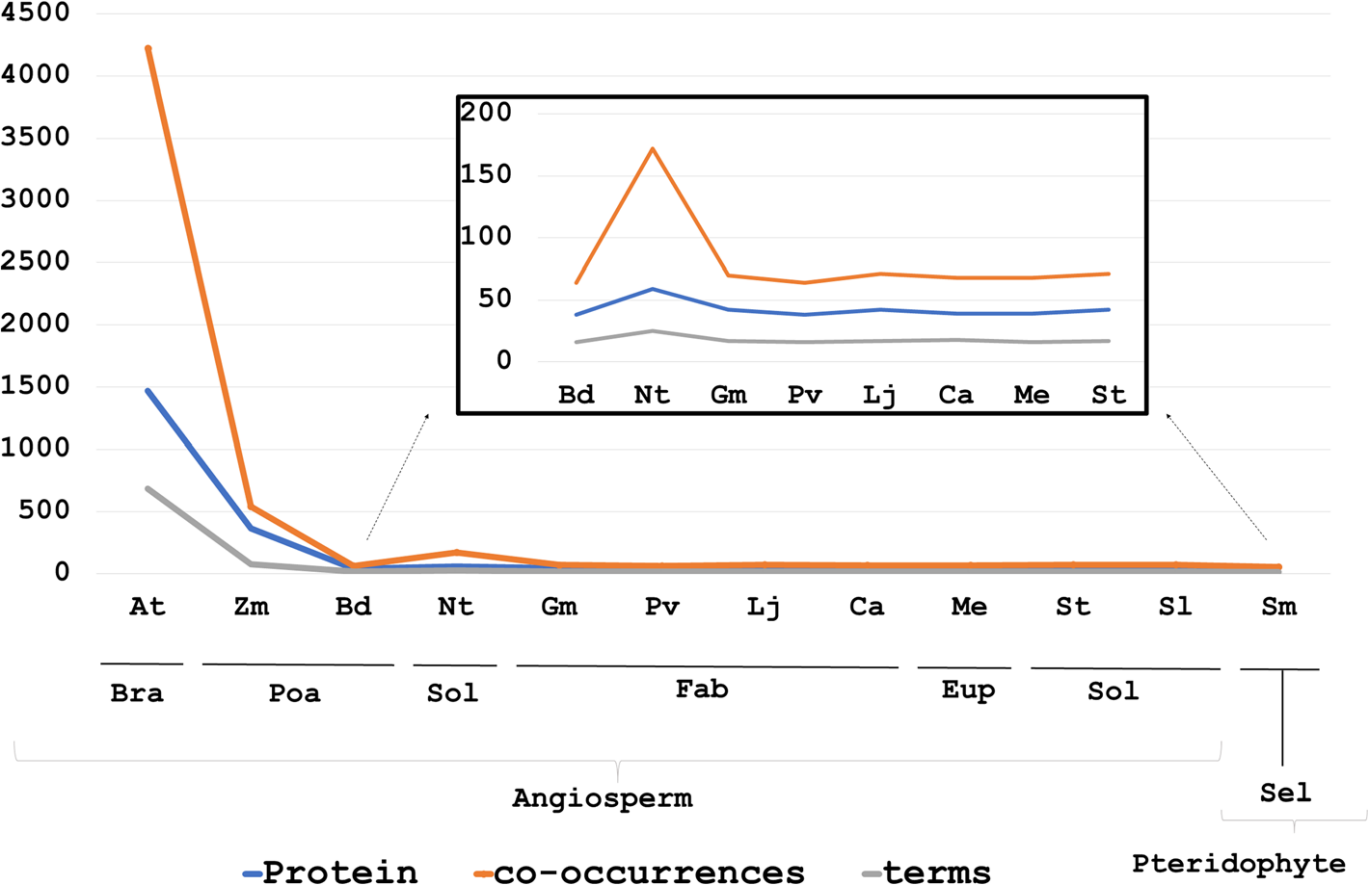
Variety of proteins, co-occurrences, and terms per species. At Y axis is possible to see the amount of each entity (proteins, co-occurrences, terms) and at X axis, species are named with the initials: **Angiosperm**: At - *Arabidopsis thaliana* (Brassicaceae); Zm - *Zea mays* and Bd - *Brachypodium distachyon* (Poaceae); Nt- *Nicotiana tabacum,* St- *Solanum tuberosum* and Sl - *Solanum lycopersicum* (Solanaceae); Gm-*Glycine max*, Pv-*Phaseolus vulgaris,* Lj-*Lotus japonicus*, Ca-*Cicer arietinum*, Me-*Manihot esculenta* (Fabaceae); **Pteridophyte**: *Selaginella moellendorffii* (Selaginellaceae). The list with all proteins, co-occurrences, and terms for each plant species is available in Supplementary Material 5

**Fig. 6:**
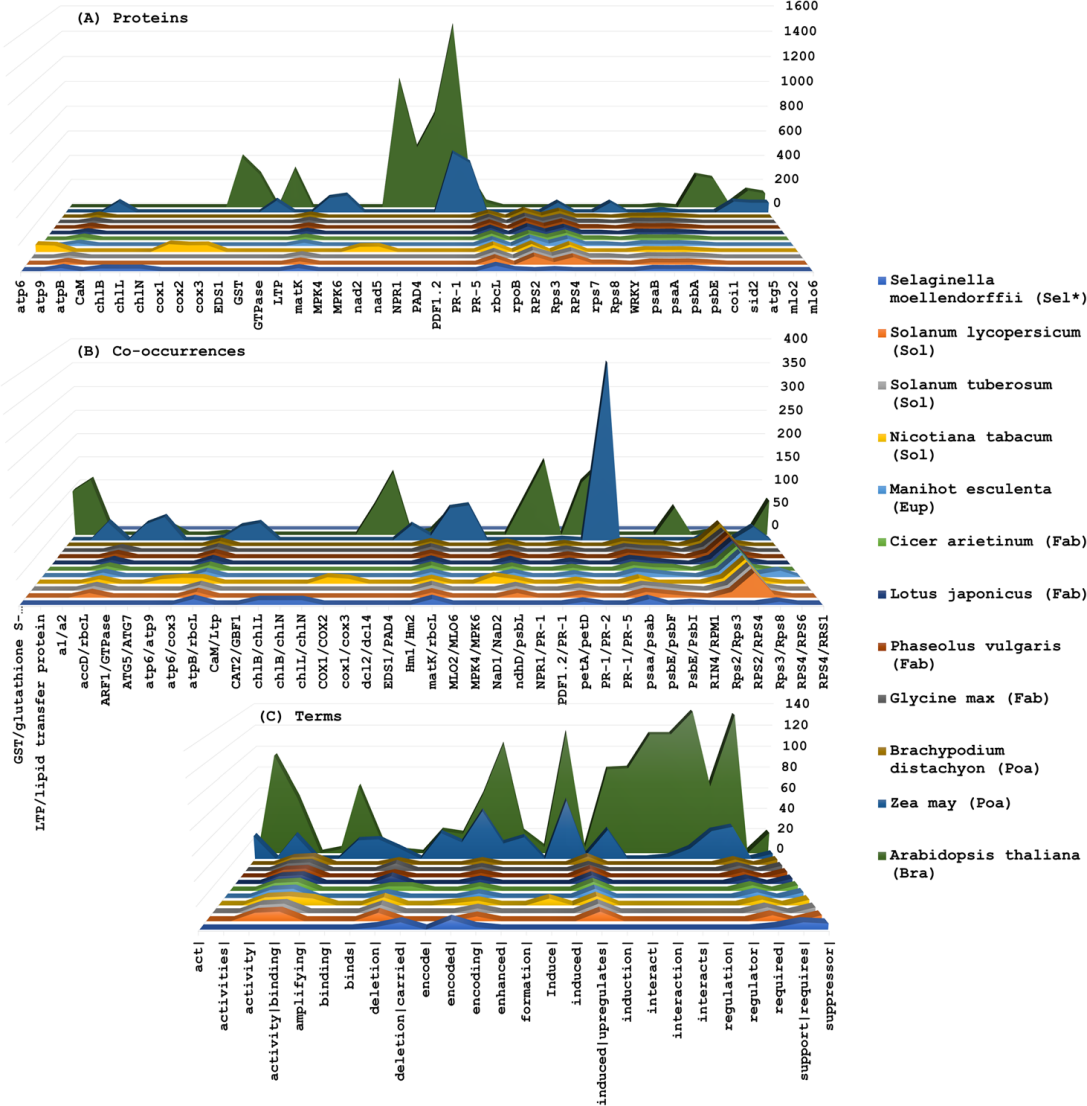
Comparison of 10 most identified proteins, co-occurrences, and terms for 12 plant species. Those species were analyzed regarding the biotic stress subset of abstracts. At Y axis the amount of each entity (proteins, co-occurrences, terms), at Z species are specifies by colors and at X-axis the entities for each item. **a** A total of 41 proteins are displayed with respective amounts. **b** A total of 38 co-occurrences can be observed with the respective number of occurrences. **c** A total of 26 interaction terms are shown with their respective quantities. **Pteridophyte**: *Selaginella moellendorffii* (Selaginellaceae). **Angiosperm**: *Solanum lycopersicum*, *Solanum tuberosum* and *Nicotiana tabacum* (Solanaceae); *Manihot esculenta* (Euphorbiaceae); *Cicer arietinum*, *Lotus japonicus*, *Phaseolus vulgaris* and *Glycine max* (Fabaceae); *Brachypodium distachyon* and *Zea mays* (Poaceae); *Arabidopsis thaliana* (Brassicaceae)

*A. thaliana* and *Zea mays* are, by far, the most studied plants (Fig. 5). Using LAITOR tax-ID against the same abstract set to filter both species interactions, a total of 14,411 and 2744 co-occurrences were mapped respectively, considering all four types. Conspicuously, *A. thaliana* registered 1468 non-redundant tagged proteins, comprised of 4224 unique co-occurrences (Fig. 6a), where PR-1 alone accounted for 1470 occurrences, highlighted in four out of 10 most abundant co-occurrences (Fig. 6b). Additionally, PR-1 was present in a total of 115 co-occurrences in the selected corpus. Terms such as *regulation* (135), *induced* (116) and *enhanced* (105) are among the most traced (Fig. 6c).

Considering *Z. mays* interactions, far fewer proteins were tagged (i.e. 365, which only represent one fourth when compared to *A. thaliana*’s amount of tagged proteins). On the other hand, *Z. mays* registered almost half the number of unique co-occurrences (2742). Therefore, a more efficient network link was displayed with PR-1, as well as with the most abundant protein, totaling 486 tagged PR-1 in 32 co-occurrences. Even though PR-1 appears only once in the 10 most cited, the interaction between PR-1 and PR-5 was registered 370 times (Fig. 6b).

In total, 38 co-occurrences were clustered, considering the 10 most relevant results for each species (Fig. 6b). Nine of those were exclusively described in *A. thaliana* (dcL2/dcL4; EDS1/PAD4; GST/glutathione S-transferase; LTP/lipid transfer protein; NPR1/PR-1; PDF1.2/PR-1; PR-1/PR-2; RIN4/RPM1; RPS4/RRS1), seven were unique in *Z. mays* (A1/A2; ARF1/GTPase; CaM/Ltp; CAT2/GBF1; Hm1/Hm2; MLO2/MLO6; MPK4/MPK6) and five were observed only in *Nicotiana tabacum* (ATP6/ATP9; ATP6/cox3; cox1/cox2; cox1/cox3; NaD1/NaD2). The interaction between matK/rbcL was the only one registered for all 12 species with similar values and, considering it regards a conserved chloroplast function, it was expected to be found in all plants. The RPS2/RPS4 co-occurrence was described for all the angiosperms searched. The pteridophyte *Selaginella moellendorffii* was the only species which did not show any RPS2/RPS4 (Fig. 6b), even after a new online keyword search on the updated 2019 MEDLINE database was performed. Despite being the least studied of all plants in the selected set, *S. moellendorffii* presents three exclusive interactions: chlB/chlL; chlB/chlN; chlL/chlN.

The other eight species (*Manihot esculenta, Cicer arietinum, Lotus japonicus, Phaseolus vulgaris, Glycine max, Brachypodium distachyon, Solanum lycopersicum, Solanum tuberosum*) revealed very similar profiles (Fig. 6). This can be explained by poorly described abstracts, missing information, or it could be due to abstracts citing more than a single species, thus causing ambiguous tagging during the NLProt process. On average, all plants have 18% of poorly studied co-occurrences (with only one or two co-occurrences registered) (Supplementary Material 5). Despite the distinct high amount of studies in *A. thaliana,* 19% of the characterized co-occurrences were poorly studied. On the other hand, only 10% of *Z. mays*’ co-occurrences were considered poorly studied, a result following the inference of efficient network construction.

### Building pathways

**Fig. 7:**
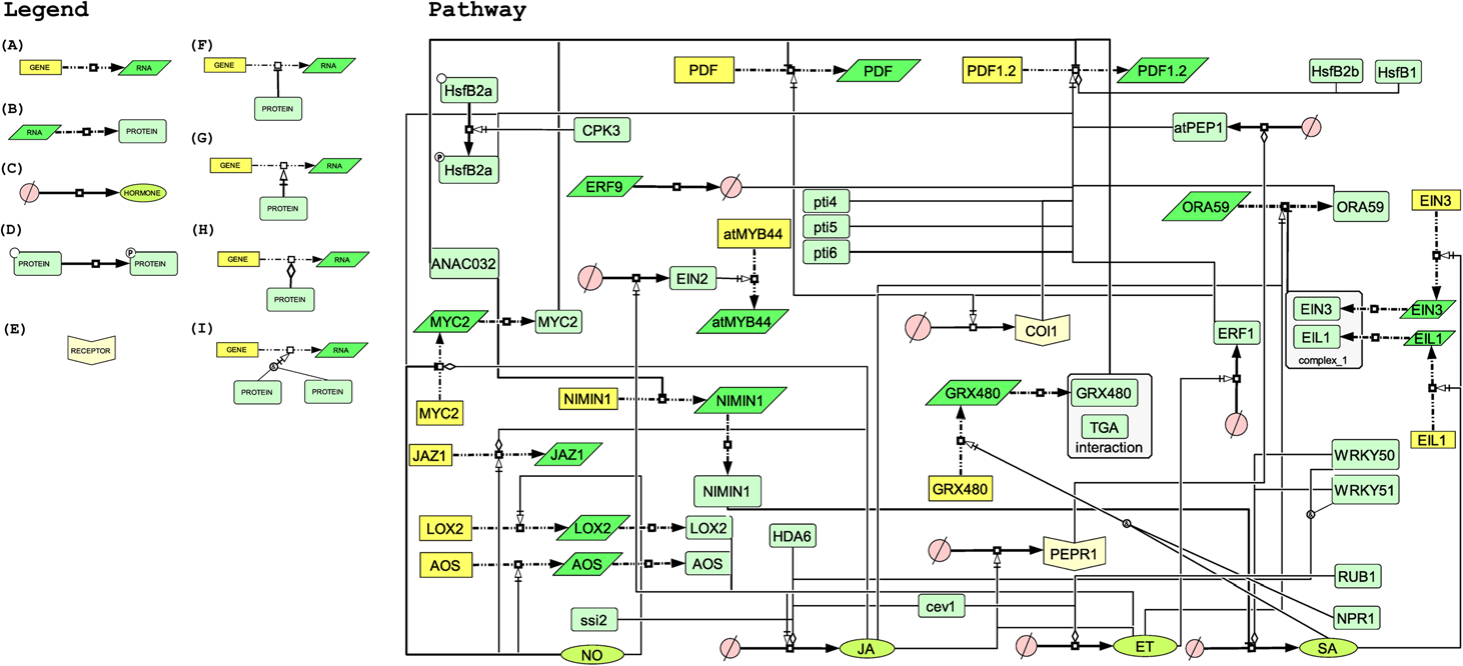
*Arabidopsis thaliana*’s defensin (PDF) pathway. On the left a representation of all interaction types recovered in the present case study: **a** Transcription; **b** Translation; **c** State transition; **d** Phosphorylation; **e** Receptor; **f** Inhibition; **g** Activation; **h** Modulation; **i** Boolean sign “&” indicating that both proteins activate a transcription. The PDF gene pathway represents the co-occurrences recovered by LAITOR4HPC when using the terms “Plant AND Defensin”. The presented data regards the summary of 31 curated abstracts containing type 1 interactions. Yellow rectangles represent genes; Bright green parallelograms represent RNA transcripts; Light green rectangles are proteins; Light yellow arrow-like figures represent a receptor; Light pink circles are inactive hormone forms; Green oval-shaped forms regard the active form of each hormone. Abbreviations: PDF: Plant Defensin; NO: nitric oxide; JA: jasmonic acid; ET: ethylene; SA: salicylic acid

The pathway annotation or enrichment is a challenging task in many aspects, mainly because it requires great efforts in the selection, examination and extraction of relevant information in the retrieved literature. This work can be even harder to be enriched or designed, depending on how large the pathways are [9], since a simple pathway can display many complex interactions (Fig. 7).

Considering the whole set retrieved by LAITOR4HPC, we selected 31 abstracts with type 1 only interactions to build the plant defensin pathway regulation in *A. thaliana* (PDF). It is important to highlight that the whole MEDLINE database was used as a training set, to tag the interaction terms and targets (e.g., genes, proteins) more efficiently. Thus, our pipeline was able to find feasible connections in 24 manuscripts that served to both automatic and manual annotation (Supplementary Material 6). To our knowledge, this is the first attempt at gathering information on a PDF network, focusing on building a pathway and specifying the relations among the entities. However, it must be mentioned that the gene encoding PDF has been tagged on MAPK signaling pathway at KEGG database (Entry ko04016). A correlation with proteins has also been reported on STRING, as, for instance, Octadecanoid-Responsive Arabidopsis (ORA59) and NPR1, both transcriptional activators [29, 30], which have as well been included in the present pathway.

The modeled pathway (Fig. 7, SMBL file available in Supplementary Material 7) indicates some well studied *A. thaliana* genes related to defense transcription factors. All tagged proteins and genes (except for PDF) either belong to TF class or are signaling regulators, like *coi1* and *pepr1* [31, 32]. A general overview of the defensin regulation pathway in *A. thaliana* allows the division of its whole structure into three main groups: signaling, regulation factors and defense response itself. For the signaling group, three hormones play a role as positive effectors: nitric oxide (NO), jasmonic acid (JA) and ethylene (ET) [33]. The second group (regulation factors) regards the transcription factors and receptors, and the third group (defense response) regards the PDF genes (Fig. 7).

### Scalability

**Fig. 8:**
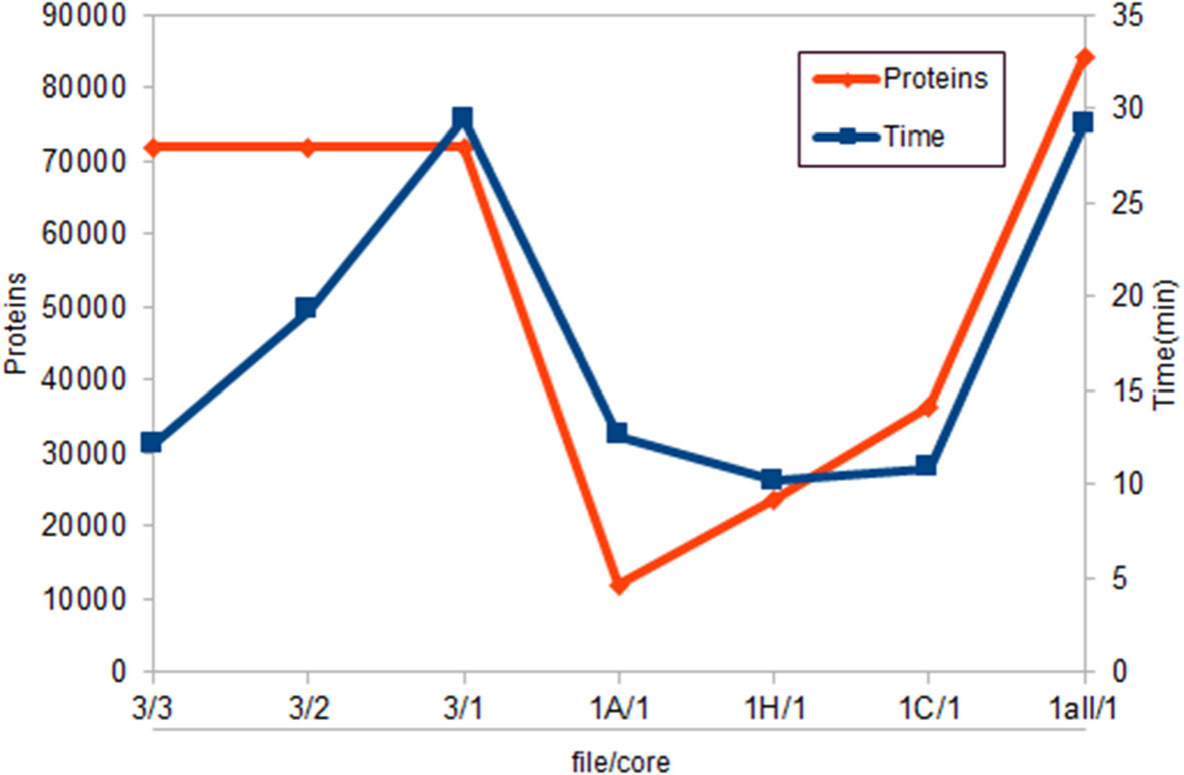
Relation among number of proteins, running time and file used to run the NlProt. 1A - *Arabidopsis thaliana;* 1H - *Homo sapiens;* 1C - *Caenorhabditis elegans;* 1all - all the abstracts gathered in one file

In the NLProt parallelized analysis, the run-time varied from 12 min (three cores and three files) to 29 min (one core and three files), tagging 71,969 proteins considering all results. For the non-parallelized analysis, the run-time varied from 10 min to 29 min for the largest file. However, on the file containing all the 3000 abstracts, the number of tagged proteins was 84,284, due to the SVM optimization performed by NLProt (Fig. 8).

**Fig. 9:**
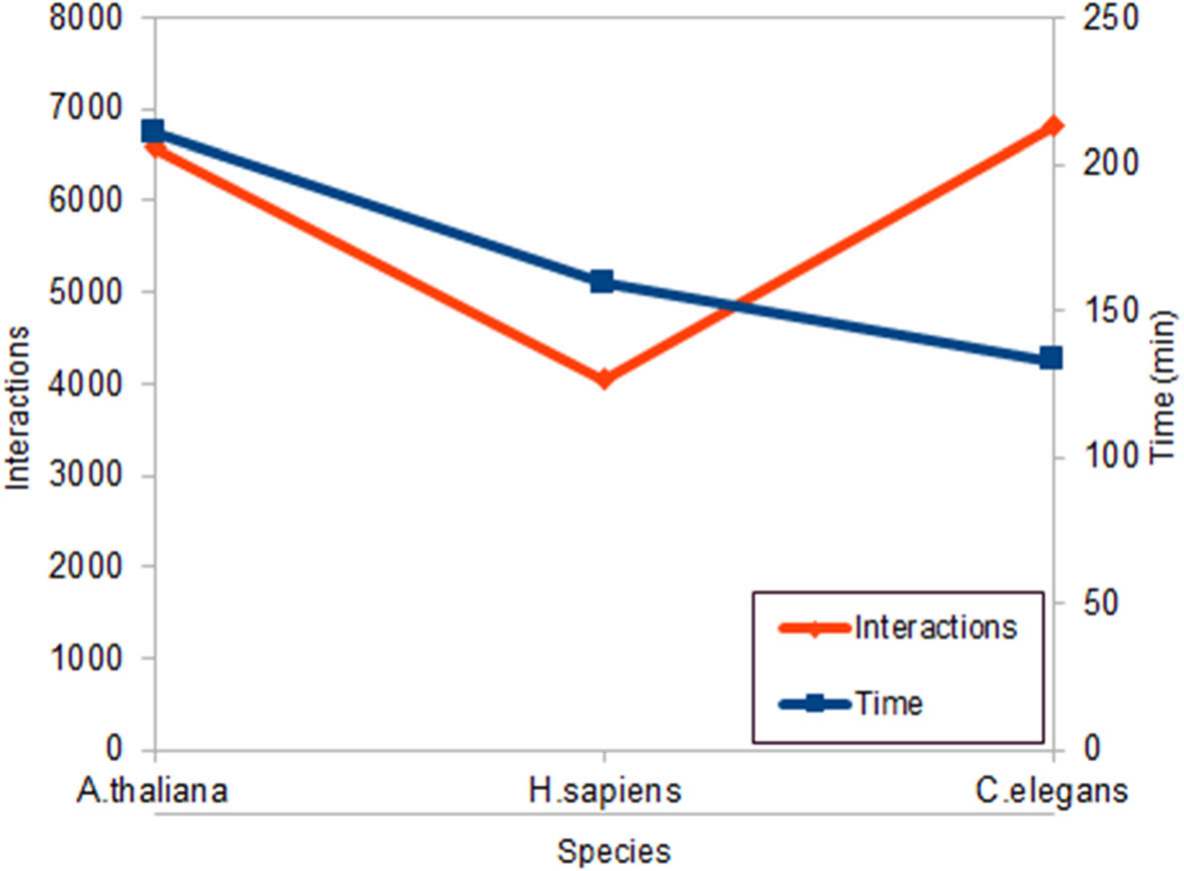
Relation among the identified interactions, running time and the species for each set of abstracts

For the LAITOR4HPC the number of interactions/running time varied from approximately 4000 interactions in 159 min for *H. sapiens* to approximately 6000 interactions in 132 min for *C. elegans*. The fact that the worm had more proteins and interactions validated in less time is due to the number of proteins and redundancies retrieved in other organisms in its abstracts set (Fig. 9).

## Discussion

The first case study identified 96 non-redundant proteins responsible for 1254 different co-occurrences in soybean, in which chloroplast-encoded protein are abundant. Besides their functional importance, chloroplast-encoded proteins are, together, widely used as a barcode for species and population studies in Fabaceae [34, 35]. As a consequence, it is not by chance that the most abundant interaction terms were: *encoding*, *amplified* and *encode*. Additionally, all top 10 co-occurrences in soybean are somehow related to photosystem II, whose proteins are part of a thylakoid structure and can be affected by high salt levels. Plants that can avoid a decrease in such proteins during stress may tolerate the stress with higher success [36]. Photosystem II proteins’ efficiency can also limit biomass [37]. For that reason, improving these proteins on plants of agronomical importance, such as soybean, is of great interest.

Fifteen species were selected, however, three had no co-occurrences described, probably due to one of the two reasons: either (1) there is no description available for the searched protein interactions, or (2) the protein interactions are not well described in the paper's abstract. Such a flaw could lead to a false-negative result, since the main text mining tools, such as LAITOR4HPC, PESCADOR [9], STRING [14–16], iHop [38], only access the abstracts. The preference for accessing abstracts is due to the difficulties in parsing full paper texts, which can include images and tables. The more objective the abstract is, including the key information, the more efficient the text mining tool will be in retrieving the results.

Considering the remaining 12 plants from the original set, PR-1 and NPR-1 proteins are the most described bioentites. The former was identified in the 1970s, and it still is largely studied and vastly induced during plant defense response. Since there is also evidence of PR-1 activity in growth and development besides stress response, its full biological role has not been completely clarified. As a consequence, the number of studies on this behalf keeps growing [39]. On the other hand, NPR-1 is a transcriptional regulator of plant stress response that is regulated by stress-released hormones recognized by plant receptors [40]. The two most representative interactions were PR-1/PR-5 and RPS2/RPS4, which contain the PR and RPS plant-disease resistance (*R*) genes, with a specific bacterial-resistant response [41, 42]. Together with the most annotated terms (*required*, *induced*, *induction*, *enhanced*), this suggests that expression profile is one of the most studied topics for plant biotic stress response.

First suggested as a model plant in 1943, *A. thaliana* has been studied for approximately 70 years, cited in more than 54,000 manuscripts until 2016 and considered a benchmark on the understanding of plant-pathogen responses, helping to enlight higher plants research. Nevertheless, this model is still an important source to fully understand stress response in flowering plants, considered an entry point for elucidating or identifying still uncovered plant-protein interaction [43–46]. Thus, it is not a surprise that this small plant stood out as the most researched plant and that the not yet completely clarified PR-1 protein is being exhaustively studied in the model plant as well.

Despite the abundance of repetitive sequences and complex genome, *Z. mays* was the second in the number of available data, exhibiting fewer tagged proteins, but almost half of the unique co-occurrences when compared with *A. thaliana*; therefore, displaying a more efficient network link. *Z. mays* is one of the primary sources for food security and one of the most studied plants when it comes to breeding studies aiming to boost productivity, seed protein quality and, especially, to raise resistance to pathogens [46–48]. Therefore, it is not a coincidence that, as in *A. thaliana*, PR-1 is also the most abundant protein in *Z. mays*.

In general, plant hormones are involved in a wide range of defense-related signaling pathways [44]. In the presented case study, four manually curated hormones (NO, JA, ET and salicylic acid; SA) interact regulating defense response. The JA hormone works like a positive effector, by activating regulation factors as COI1 and PEPR1, which is also activated by ET. In turn, PEPR1 modulates at PEP1 (as COI1), involved in *PDF1.2* transcription induction [31, 45, 46]. Additionally, JA controls defensin expression by inducing specific transcription factors, as ORA59 [29], or by modulating the transcription of *MYC2* that inhibits PDF [47].

Another signaling molecule that plays an essential role in the pathway is NO, first because it inhibits *MYC2* transcription and, second, because it induces *PDF* expression. Besides, NO also activates the JA signaling positive effectors *LOX2* and *AOS* [47]. Thus, it plays a role in the pathway, not only by enabling transcription factors to induce the defense response, but also by regulating JA signaling intensity. Finally, ET signaling hormone can induce *PDF* transcription indirectly by activating ORA59 and ERF1 [29, 46] (Fig. 7). Both ORA59 and ERF1 are positive effectors to *PDF1.2* transcription, despite activating PEPR1 receptor, an indirect positive regulator of defensin transcription, as aforementioned [31].

The only hormone mapped as a negative regulator of *A. thaliana* defensin expression was SA, by inducing the *EIN3* and *EIL1* transcription, which become a complex EIN3/EIL1. This complex is responsible for inhibiting the positive regulator ORA59 [29] as mentioned before. Additionally, SA hormone and NPR1 protein activate *GRX480* transcription. Once active, it forms an interaction complex with TGA and inhibits *PDF1.2* transcription [48] (Fig. 7). These results show how complex the plant defense regulation can be and shed some light to understand the cross factors that may occur. Thus, in this case study, the pipeline was very effective, not only in retrieving information automatically, but also in providing a significant and pertinent abstract set for manual annotation. Such a combination of approaches allowed specifying the correlation among the entities in an efficient way, giving a more detailed view of the defensin regulation pathway in *A. thaliana*.

Most of the current text mining tools are either online, like PESCADOR [9], STRING [14–16] and PPIcurator [49]; or very specific, such as FamPlex [50], for human proteins, MPTM [51], for post-translation modification in humans, and PaperBLAST [52], for homology search. LAITOR4HPC and STRING updates are the only programmatic text mining tools available that came to our knowledge. Nevertheless, both have different approaches. STRING focuses on co-occurrences within neighborhood genes and uses protein names for keyword searches. Text mining functionality is directed to corroborate interactions in their database and, when used separately, it retrieves only the top tagged proteins [14–16]. On the other hand, LAITOR4HPC pipeline is intended to retrieve information from a different point of view, thus providing flexibility in research topics. Our pipeline is prepared to search all the interactions of a given PubMed XML corpus, retrieving data for a comprehensive network design. Besides, LAITOR4HPC can help spotting co-occurrences that have already been exhaustively studied, as well as highlight some that have been poorly studied or that still have not been considered.

The parallelization has sped up the analyses, since it avoids the pilling up of files. The time rate comparison revealed a speed improvement of more than double from the parallelized version to the non-parallelized one.

## Conclusion

The improvement of LAITOR [8] and development of LAITOR4HPC has decreased computing time significantly, due to the implementation of parallelization. Such an increase resulted not only in much faster run time, but also maintained the consistency and reliability of previously LAITOR implementations. Time will vary accordingly, depending on available hardware resources, specially regarding memory capacity and the number of available cores. Since this improved online tool includes only data from abstracts, it is essential to consider manual data curation to confirm predicted protein-protein interactions from co-occurrences terms.

Despite its economic importance and intensive research investments, most soybean publications are focused on chloroplast-encoded proteins, rather than on stress-responsive proteins. On the other hand, in the case study that analyzed the biotic stress terms, PR-1 was the most representative protein, and probably some effort should be applied to clarify other genes/proteins related to the biotic stress response. A more comprehensive subset of described interactions can fill gaps in the understanding of PR-1 role and in other relevant pathways related to the biotic stress response. Using manual and automatic annotation, the pipeline provided a very detailed pathway with literature support, evidencing the components of plant defensin signalling and modulation. Thus, it maintains the accuracy of PESCADOR with the improved possibility to analyze big data in a short time.

LAITOR4HPC is suitable for establishing or enriching new interaction pathways. It has shown to be efficient in retrieving reliable information, providing an overview for a given target, or even for a given keyword associated with an organism of interest. It is important to highlight that the pipeline was able to retrieve all the relevant sets of papers for the searched topics in a more efficient way than just digging into the list of MEDLINE publications. Since the number of manuscripts is increasing quickly, new approaches for linking information are demanded to enable a fast, reliable and prompt way of fully understanding the targeted taxon's or organism's systems biology.

As take-home message, for more efficient development and application of tools, such as LAITOR4HPC in Systems Biology, future publications should include some ‘minimal information about publication of interaction data’ (MIAPID) preferably in a tabular format. This summary of identified and validated interactions will simplify the data recovery and integration to generate or enrich existing pathways.

### Datasets availability

**Project name:**LAITOR4HPC

**Project home page:** https://zenodo.org/record/1717329

**Operating system(s): e.g.,** Linux (Ubuntu 16.04+) and macOS 10+

**Programming language:** Python v.3, PHP v.7

**Other requirements:** Perl v.5.22, SQLite v.3

**License**: GNU Affero General Public License (AGPL 3.0)

**Any restrictions to use by non-academics:** No restriction

## Supplementary information

**Additional file 1.** PMID list. Unformatted text file containing the list of PMIDs used to the soybean analysis. The list represents all the MEDLINE PMIDs available until July 2017.

**Additional file 2. **Searching bioentities co-occurrences in all available abstracts for one specie (First case study report). Excel file containing information about the total of proteins, co-occurrences and terms of interaction mapped by LAITOR4HPC in *Glicine max*. For each category there are information about the absolute number of each element category, the total number of elements and the number of non-redundant elements. Additionally, one section is dedicated to the total for each interaction type, INT_1 (more likely to be effective) to INT_4 (less likely to be effective).

**Additional file 3. **Keywords. PDF file listing the 20 keywords used to filter PMID related to biotic stress on case study 3. Each keyword was run on LAITOR4HPC 15 times, one time for each plant species tax-ID (*Arabidopsis thaliana*, *Zea mays*, *Brachypodium distachyon, Nicotiana tabacum, Solanum tuberosum, Solanum lycopersicum,* Gm-*Glycine max, Phaseolus vulgaris, Lotus japonicus*, *Cicer arietinum*, *Manihot esculenta, Selaginella moellendorffii, Medicago truncatula, Nicotiana benthamiana,* and *Ricinus communis*)*.*

**Additional file 4.** Using keywords to look for all described interactions on one subject, all plants summary (Second case study report). Excel file containing information about the total of proteins, co-occurrences and terms of interaction mapped by LAITOR4HPC in 12 plant species considering the biotic stress related PMIDs. For each category there are information about the absolute number of each element category, the total number of elements and the number of non-redundant elements. Additionally, one section is dedicated to the total for each interaction type, INT_1 (more likely to be effective) to INT_4 (less likely to be effective).

**Additional file 5. **Using keywords to look for all described interactions in *Arabidopsis thaliana* (Third case study report). Excel file containing information about the total of proteins, co-occurrences and terms of interaction mapped by LAITOR4HPC in *Arabidopsis thaliana*. For each category, there are information about the absolute number of each element category, the total number of elements and the number of non-redundant elements. Additionally, one section is dedicated to the total for each interaction type, INT_1 (more likely to be effective) to INT_4 (less likely to be effective).

**Additional file 6. **Building pathways (Third case study report). Excel file containing a curated list of interaction identified with LAITOR4HPC in *Arabidopsis thaliana* for the “plant AND defensing” keywords filtered PMIDs. This list embraces automatic and manual annotation of the mapped data.

**Additional file 7. ***Arabidopsis thaliana pathway.* The SMBL file is a xml-based file format storing the computational biological model of the pathway. The file contains the defensing interaction network of *Arabidopsis thaliana*, and is compatible with CellDesigner tool for biological network visualization and creation.

## Data Availability

The datasets analyzed for this study can be found in Zenodo repository [zenodo.org], as well as the files required for LAITOR4HPC installing process. The dataset can be found by searching for a repository called LAITOR4HPC **[**https://zenodo.org/record/1717329#.XAXWpmhKhPY**]** and is registered at DOI: 10.5281/zenodo.1717329. All datasets generated and analyzed for this study are included in the manuscript and the supplementary files.

## Abbreviations

Symbol: Abbreviation meaning
A1: Dihydroflavonol 4-reductase
A2: Leucoanthocyanidin dioxygenase
AOS: Allene oxide synthase
ARF: Auxin Response Factors
ATP: Adenosine Triphosphate
CaM: Calmodulin
CAT: Catalase
chl: Chloroplast
coi: Coronatine Insensitive
cox: Cytochrome oxidase
DB: Database
dcL: Dicer-like
EDS: Enhanced disease susceptibility
EIL: Ethylene Insensitive-Like
EIN: Ethylene Insensitive
ERF: Ethylene Response Factor
ET: Ethylene
GBF: G-Box Factors
GRX: Glutaredoxin
GST: Glutatione s-transferase
Hm1: NADPH HC toxin reductase
Hm2: *Helminthosporium carbonum* susceptibility
HPC: High Performance Computing
IR: Information Retrival
JA: Jasmonic acid
LOX: Lypoxigenase
LTP: Lipid transfer protein
MAPK: Mithogen activated protein kinase
matK: Maturase K
MLO: Mildew resistance Locus
MPK: Mitogen-Activated Protein Kinase
MYC: Jasmonate Insensitive
NaD: *Nicotiana alata* Defensin
NCBI: National Center for Biotechnology Information
NO: Nitric oxide
NPR: Noneexpressor of Pathogenesis-related Gene
ORA59: Octadecanoid-Responsive Arabidopsis
PAD: Phytoalexin Deficient
PDF: Plant Defensin
pepr: Perception of the Arabidopsis Danger Signal Peptide
PMID: PubMed ID
PR: Pathogenesis-related
psb: Photosystem II protein
rbcL: Rubisco Large Subunit
RIN: RPM-Interacting Protein
RPM1: *Pseudomonas syringae pv maculicola*1
RPS: Ribosomal protein small subunit
SA: Salicylic acid
SCP: Secure copy protocol
SSH: Secure Shell
tax-ID: Taxonomy ID (NCBI)
TGA: Transcription Factor TGA
