# Supplementary material for: LAITOR4HPC: A text mining pipeline based on HPC for building interaction networks
Source: BMC Bioinformatics. 2020 Aug 24;21:365. doi: 10.1186/s12859-020-03620-4 (PMC7447576; doi:10.1186/s12859-020-03620-4)
Supplement: Supplementary file 3 — Additional file 3. Keywords. PDF file listing the 20 keywords used to filter PMID related to biotic stress on case study 3. Each keyword was run on LAITOR4HPC 15 times, one time for each plant species tax-ID (Arabidopsis thaliana, Zea mays, Brachypodium distachyon, Nicotiana tabacum, Solanum tuberosum, Solanum lycopersicum, Gm-Glycine max, Phaseolus vulgaris, Lotus japonicus, Cicer arietinum, Manihot esculenta, Selaginella moellendorffii, Medicago truncatula, Nicotiana benthamiana, and Ricinus communis). [file 12859_2020_3620_MOESM3_ESM.pdf]

	Keywords
1	pr AND plant
2	ltp AND plant
3	amp AND plant
4	virus AND plant
5	nbs-lrr AND plant
6	hevein AND plant
7	fungus AND plant
8	snakin AND plant
9	thionin AND plant
10	knottin AND plant
11	defense AND plant
12	defensin AND plant
13	cyclotide AND plant
14	pathogen AND plant
15	resistance AND plant
16	thaumatin-like protein AND plant
17	protein interaction AND plant disease
18	protein interaction AND plant infection
19	protein interaction AND plant biotic stress
20	protein interaction AND plant pathogen interaction
